# Helminth infections and gut microbiota – a feline perspective

**DOI:** 10.1186/s13071-016-1908-4

**Published:** 2016-12-03

**Authors:** Ana M. Duarte, Timothy P. Jenkins, Maria S. Latrofa, Alessio Giannelli, Elias Papadopoulos, Luís Madeira de Carvalho, Matthew J. Nolan, Domenico Otranto, Cinzia Cantacessi

**Affiliations:** 1 Department of Veterinary Medicine, University of Cambridge, Cambridge, UK; 2 CIISA, Faculty of Veterinary Medicine, Universidade de Lisboa, Lisboa, Portugal; 3 Department of Veterinary Medicine, University of Bari, Valenzano, Italy; 4 School of Veterinary Medicine, Faculty of Health Sciences, Aristotle University of Thessaloniki, Thessaloniki, Greece; 5 Department of Pathology and Pathogen Biology, Royal Veterinary College, University of London, Hatfield, UK

**Keywords:** Gut microbiota, Cat, *Toxocara cati*, Lactobacilli, Microbial richness and diversity, 16S rRNA

## Abstract

**Background:**

Investigations of the relationships between the gut microbiota and gastrointestinal parasitic nematodes are attracting growing interest by the scientific community, driven by the need to better understand the contribution of parasite-associated changes in the composition of the gut flora to both host malnutrition and immune modulation. These studies have however been carried out mainly in humans and experimental animals, while knowledge of the make-up of the gut commensal flora in presence or absence of infection by parasitic nematodes in domestic animals is limited. In this study, we investigate the qualitative and quantitative impact that infections by a widespread parasite of cats (i.e. *Toxocara cati*) exert on the gut microbiota of feline hosts.

**Methods:**

The faecal microbiota of cats with patent infection by *T. cati* (= *Tc*+), as well as that of negative controls (= *Tc*-) was examined via high-throughput sequencing of the V3-V4 hypervariable region of the bacterial 16S rRNA gene, followed by bioinformatics and biostatistical analyses of sequence data.

**Results:**

A total of 2,325,366 useable high-quality sequences were generated from the faecal samples analysed in this study and subjected to further bioinformatics analyses, which led to the identification of 128 OTUs and nine bacterial phyla, respectively. The phylum Firmicutes was predominant in all samples analysed (mean of 53.0%), followed by the phyla Proteobacteria (13.8%), Actinobacteria (13.7%) and Bacteroidetes (10.1%). Among others, bacteria of the order Lactobacillales, the family *Enterococcaceae* and genera *Enterococcus* and *Dorea* showed a trend towards increased abundance in *Tc*+  compared with *Tc*- samples, while no significant differences in OTU richness and diversity were recorded between *Tc*+ and *Tc*- samples (*P* = 0.485 and *P* = 0.581, respectively). However, Canonical Correlation and Redundancy Analyses were able to separate samples by infection status (*P* = 0.030 and *P* = 0.015, respectively), which suggests a correlation between the latter and the composition of the feline faecal microbiota.

**Conclusions:**

In spite of the relatively small number of samples analysed, subtle differences in the composition of the gut microbiota of *Tc*+ *vs Tc*- cats could be identified, some of which in accordance with current data from humans and laboratory animal hosts. Nevertheless, the findings from this study contribute valuable knowledge to the yet little explored area of parasite-microbiota interactions in domestic animals.

**Electronic supplementary material:**

The online version of this article (doi:10.1186/s13071-016-1908-4) contains supplementary material, which is available to authorized users.

## Background

The gastrointestinal (GI) tract of humans and animals is inhabited by a myriad of microorganisms, the gut microbiota, which are essential for the maintenance of the homeostasis of the digestive system [1, 2] and whose functions span nutrition and metabolism, protection against pathogens and regulation of both innate and adaptive immune responses [1, 2]. In parallel, a number of nematode parasites of vertebrates have developed, over millions of years of evolution, a range of strategies to evade or dampen host immunity, thus providing them with the ability to chronically colonise the GI tract of humans and animals [3]. It is therefore plausible that the successful establishment of parasitic nematodes in the vertebrate gut is achieved, at least in part and directly and/or indirectly, via physical, molecular and/or immunological interactions with the resident commensal flora [3, 4]. Indeed, over the past few years, investigations of the relationships between the gut microbiota and parasitic nematodes have attracted growing interest by the scientific community, mainly driven by the need to better understand the contribution of parasite-associated changes in the composition of microbial populations to host malnutrition [4]. In addition, a number of studies have focused on the immune-modulatory properties shared by both commensal bacteria and GI parasitic nematodes, in a bid to address questions on the possible role/s of helminth-induced fluctuations in gut microbiota in parasite-driven suppression of inflammation [3, 5, 6]. However, these studies have been carried out in a limited range of vertebrate hosts and for a few species of GI nematodes; these include humans experimentally infected with hookworms (i.e. *Necator americanus*) [7, 8] or naturally infected with hookworms and/or whipworms (*Trichuris* spp.) and/or roundworms (*Ascaris* spp.) [9, 10], and laboratory animals infected with strains of *Heligmosomoides polygyrus bakeri*, *Trichuris muris* [11–15] or *T. suis* [16]. The specific findings from these studies differ considerably, with some pointing towards an overall increase in microbial species richness and diversity in response to nematode infection [7, 8, 10, 16] and others recording detectable shifts in the abundance of specific populations of bacteria following parasite establishment [3]. Given these inconsistencies, further studies in other host-parasite systems are required in order to determine whether changes in the composition of the commensal flora that occur in concomitance with colonisation by GI parasitic nematodes are dependent upon the animal host and/or the parasite involved and/or the burden of infection.

Domestic animals, for instance, provide useful systems for the collection of data on helminth-microbiota interactions under natural conditions, since they are often infected by a range of species of GI parasitic nematodes (i.e. enoplids, strongylids and ascarids) and by varying parasite loads [17–20]. However, thus far, only a handful of studies have explored the relationships between GI enoplids and strongylids and the commensal gut flora in non-experimental animals. These studies include recent investigations of changes in the composition of the microbiota of the proximal colon of pigs infected with *T. suis* [21], of the abomasum of goats infected with *Haemonchus contortus* [22] and of dogs infected with *Ancylostoma caninum* [23]. However, despite these efforts, knowledge of this area remains fragmentary. In addition, to the best of our knowledge, no studies have thus far investigated the relationships between ascarid parasites and the gut commensal flora. This link is of particular interest, given the known immune-modulatory properties of these large GI nematodes [24] as well as their association with the onset of allergy in at-risk populations [25] Therefore, the elucidation of the relationships between ascarids of domestic animals and their gut microbiota may provide useful information towards elucidating the relative contribution of parasite-associated changes in gut commensal microbes to host immune-modulation.

In this study, capitalising on the sampling opportunities provided by a recent clinical trial [26], we investigated the qualitative and quantitative impact that patent infections by *Toxocara cati* exert on the gut microbiota of the cat hosts.

## Methods

### Study cohorts

Cats enrolled in this study were initially selected based on the following criteria: (i) Owned and living in a relatively restricted area of Thessaloniki (Greece); (ii) Weaned; (iii) Fed an identical diet of commercial dry food (i.e. Purina Friskies®) for at least 6 months prior to sampling; (iv) Allowed to roam free in outdoor areas and hunt; (v) Clinically healthy (e.g. absence of signs of GI disease or any other concomitant disease); (vi) Not treated with antibiotics and/or anthelmintics over 12 and 3 months prior to sample collection, respectively.

Only cats with or without patent *T. cati* infection (= *Tc*+ and *Tc*-, respectively) and negative for other helminths (i.e. hookworms and tapeworms) and protozoan parasites (i.e. *Isospora* spp., *Giardia* spp. and *Cryptosporidium* spp.) at the faecal examination (see below) were included. A total number of 45 cats (*Tc*+ = 24, 7 males and 17 females; and *Tc*- = 21, 5 males, 16 females) matched these criteria (Table 1). None of the female cats included in this study was pregnant or lactating at the time of sample collection.

**Table 1:** Gender, age (months) and weight (kg) of *Toxocara cati*-positive (*Tc*+) and *T. cati*-negative (*Tc*-) cats enrolled in this study. The infection burden of *Tc*+ cats is indicated in eggs per gram of faeces (EPG)

*Tc*+					*Tc*-			
ID	Gender	Age	Weight	EPG	ID	Gender	Age	Weight
I001	F	24	> 2.5	200	C002	F	12	< 2.5
I005	M	5	< 2.5	750	C003	F	36	> 2.5
I007	F	24	> 2.5	200	C004	F	120	> 2.5
I008	M	30	> 2.5	300	C006	M	12	> 2.5
I009	M	18	–	150	C010	F	24	–
I012	F	36	> 2.5	150	C011	F	30	–
I015	F	18	> 2.5	200	C013	F	36	> 2.5
I017	F	12	< 2.5	300	C014	M	12	> 2.5
I018	F	30	> 2.5	200	C016	F	18	> 2.5
I019	F	6	< 2.5	400	C021	F	24	> 2.5
I020	F	12	> 2.5	300	C022	F	24	> 2.5
I023	M	12	> 2.5	300	C025	M	12	> 2.5
I024	F	18	> 2.5	450	C028	F	30	> 2.5
I026	F	24	> 2.5	300	C029	F	24	> 2.5
I027	F	24	> 2.5	100	C030	F	36	> 2.5
I031	F	60	> 2.5	100	C034	M	12	> 2.5
I032	F	36	> 2.5	200	C035	F	36	> 2.5
I033	F	24	> 2.5	150	C037	F	18	> 2.5
I036	F	12	> 2.5	100	C043	F	30	> 2.5
I038	M	24	> 2.5	300	C044	M	24	> 2.5
I039	M	12	> 2.5	350	C045	F	24	> 2.5
I040	F	24	> 2.5	100				
I041	F	18	> 2.5	100				
I042	M	18	> 2.5	400				

*Abbreviations*: *F* female, *M* male

### Sample collection, DNA extraction and high-throughput sequencing

Once collected, fresh faecal samples were stored in sterile tubes at room temperature, and immediately transported to the Laboratory of Parasitology and Parasitic Diseases, School of Veterinary Medicine of the Aristotle University of Thessaloniki (Greece), where they were refrigerated (at 4 °C) prior to processing. Briefly, individual samples were aliquoted for use in standard parasitological procedures, i.e. faecal egg counts (FEC) using a standard McMaster technique, as well as DNA extraction followed by high-throughput sequencing of the bacterial 16S rRNA gene (see below). For microscopical examination, aliquots of 2 g of faeces were suspended in 28 ml zinc sulphate solution (ZnSo_4_, specific gravity = 1.180); the suspension was homogenised, filtered using a double-layer gauze, and pipetted into McMaster chambers for microscopical examination. The remaining aliquots from these faecal samples (approximately 4 g for each sample) were homogenized, preserved in 70% ethanol, and shipped to the Parasitology Unit of the University of Bari (Italy), where they were kept at -80 °C, until DNA extraction.

Total genomic DNA was extracted directly from two technical replicates of each *Tc*+ and *Tc*- sample, as well as from two negative ‘blank’ (= no DNA) controls, using the PowerSoil® DNA Isolation Kit (MO BIO Laboratories, Carlsbad, CA, USA), according to the manufacturers’ instructions. High-throughput sequencing of the V3-V4 hypervariable region of the bacterial 16S rRNA gene was performed on an Illumina MiSeq platform according to the manufacturers’ protocols with minor adjustments. Briefly, the V3-V4 region was PCR amplified using universal primers (Forward, 5′-TCG TCG GCA GCG TCA GAT GTG TAT AAG AGA CAG CCT ACG GGN GGC WGC AG-3′; Reverse, 5′-GTC TCG TGG GCT CGG AGA TGT GTA TAA GAG ACA GGA CTA CHV GGG TAT CTA ATC C-3′) [27], that contained the Illumina adapter overhang nucleotide sequences, using the NEBNext® Q5® Hot Start HiFi DNA polymerase (New England Biolabs® Inc, Massachusetts, USA) and the following thermocycling protocol: 98 °C/2 min, followed by 35 cycles of 98 °C/15 s, 63 °C/30 s, and 72 °C/30 s, and a final elongation step of 72 °C/5 min. Amplicons were purified using AMPure XP PCR Purification beads (Beckman Coulter, Brea, California, USA), and the NEBNext hot start high-fidelity DNA polymerase was used for the index PCR with Nextera XT index primers (Illumina, San Diego, California, USA) according to the following thermocycling protocol: 98 °C/30 s, 8 cycles of 98 °C/10 s, 65 °C/75 s and at 65 °C/5 min. The indexed samples were purified using AMPure XP beads, quantified using the Qubit Quant-iT™ dsDNA Broad-Range Assay Kit (Life Technologies, Carlsbad, California, USA), and equal quantities from each sample pooled. The resulting pooled library was quantified using the NEBNext® Library Quant Kit for Illumina® (New England Biolabs® Inc) and sequenced on an Illumina MiSeq platform using the v3 chemistry (301 bp paired-end reads). Raw sequence data have been deposited in the NCBI Sequence Read Archive database under accession number PRJNA349988.

### Bioinformatics analyses

Raw paired-end Illumina reads were trimmed for 16S rRNA gene primer sequences using Cutadapt (https://cutadapt.readthedocs.org/en/stable/) and reads were joined using the Quantitative Insights Into Microbial Ecology (QIIME) software suite (version 1.9.0) [28]. Successfully joined sequences were quality filtered in QIIME using the usearch_qf script with default settings. Then, sequences were clustered into Operational Taxonomic Units (OTUs) on the basis of similarity to known bacterial sequences available in the Greengenes database (v13.8; http://greengenes.secondgenome.com/; 97% sequence similarity cut-off) using the UCLUST software; sequences that could not be matched to references in the Greengenes database were clustered *de novo* based on pair-wise sequence identity (97% sequence similarity cut-off). The first selected cluster seed was considered as the representative sequence of each OTU. Then, representative sequences were assigned to taxonomy using the UCLUST software. OTUs assigned to sequences obtained from the no-DNA control samples, as well as singleton OTUs, were removed prior to downstream analysis. Normalisation was carried out by generating a subsampled OTU table by random sampling (without replacement) of the input OTU table using an implementation of the Mersenne twister algorithm (http://www.numpy.org/). Subsequently, OTU tables were rarefied to accommodate for different sampling depths. Samples characterised by fewer than the requested rarefaction depth (i.e. 26,036 sequences) were omitted from the output OTU table. Statistical analyses were executed in R version 3.1.2 (http://www.r-project.org/); normality of variables was tested by Shapiro test and equality of variance by Levene test. Differences in the composition of the feline gut microbiota between *Tc*+ and *Tc-* samples were assessed using the LDA Effect Size (LEfSe) workflow [29], by assigning ‘helminth infection status’ as comparison class, and verified by paired *t*-test for taxa with normal distribution and equal variance. Differences in bacterial diversity and richness in control and infected samples were evaluated using paired *t*-test.

## Results

**Fig. 1:**
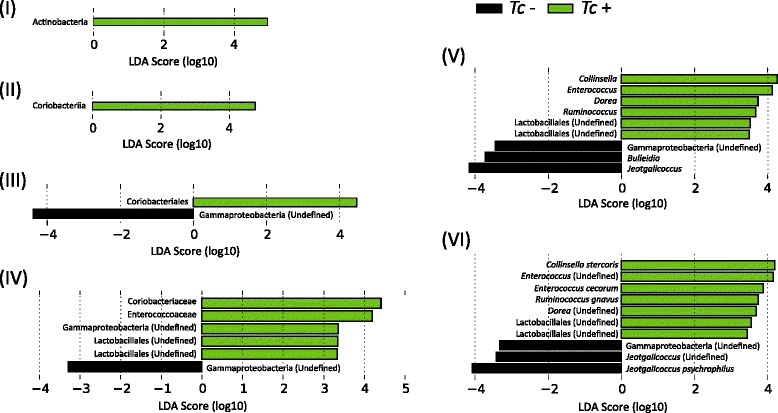
Rank plot of differentially abundant faecal bacteria (at the phylum - I, class - II, order - III, family - IV, genus - V and species - VI level) between *Toxocara cati*-positive (*Tc*+) and -negative (*Tc*-) cats, based on LDA Effect Size (LEfSe) analysis. Taxa highlighted in green/black indicate overrepresentation in *Tc*+ and *Tc*- samples, respectively

**Fig. 2:**
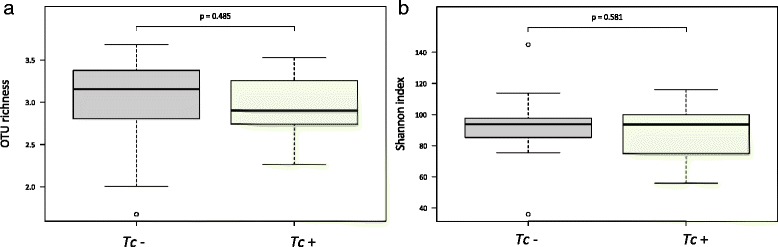
Differences in overall taxonomic species richness (**a**) and diversity (**b**) between the faecal microbiota of *Toxocara cati*-positive (*Tc*+) and *T. cati*-negative (*Tc*-) cats

A total of 7,063,321 paired-end reads were generated from the 45 samples analyzed in the present study (per sample mean 160,530 ± 76,194) (not shown). However, due to low read counts, sample I26 (i.e. 1,438 sequences) was excluded. The remaining 7,061,883 reads were subjected to further processing. Following primer trimming, joining of paired-end reads, filtering of low-quality sequences and removal of ‘contaminant’ and singleton OTUs, a total of 2,325,366 high-quality sequences were retained for further bioinformatics analyses. The rarefaction curves generated following *in silico* subtraction of low-quality and contaminant sequences indicated that the vast majority of feline faecal bacterial communities were represented in the remaining sequence data, thus allowing us to undertake further analyses. These sequences were assigned to 128 OTUs and 9 bacterial phyla, respectively (Additional file 1: Dataset S1). The composition of the faecal microbiota of *Tc*+ and *Tc*- cats is shown in Additional file 2: Figure S1. The phylum Firmicutes was predominant in all samples analysed in the present study (mean of 53.0%), followed by the phyla Protebacteria (13.8%), Actinobacteria (13.7%) and Bacteroidetes (10.1%) (Additional file 2: Figure S1). Analysis by LefSe, also supported by paired *t*-test, identified differences in abundance of individual taxa at the family, genus and species level between *Tc*+ and *Tc*- cats (Fig. 1). In particular, Actinobacteria (phylum), Coriobacteriia (class), Coriobacteriales (order), *Coriobacteriaceae*, *Enterococcaceae*, *Gammaproteobacteria* and undefined *Lactobacillales* (family), *Collinsella*, *Enterococcus*, *Dorea*, *Ruminococcus* and undefined *Lactobacillales* (genus) showed a trend towards increased abundance in *Tc*+ subjects compared with *Tc*- samples (Fig. 1). Conversely, sequences belonging to Gammaproteobacteria (order, family, genus and species) and the genera *Bulleidia* and *Jeotgalicoccus* were less abundant in *Tc*+ samples compared with *Tc*- (Fig. 1). No significant differences in OTU diversity and richness were recorded between *Tc*+ and *Tc*- samples (Shannon index, *P* = 0.581 and richness, *P* = 0.485, respectively) (Fig. 2).

**Fig. 3:**
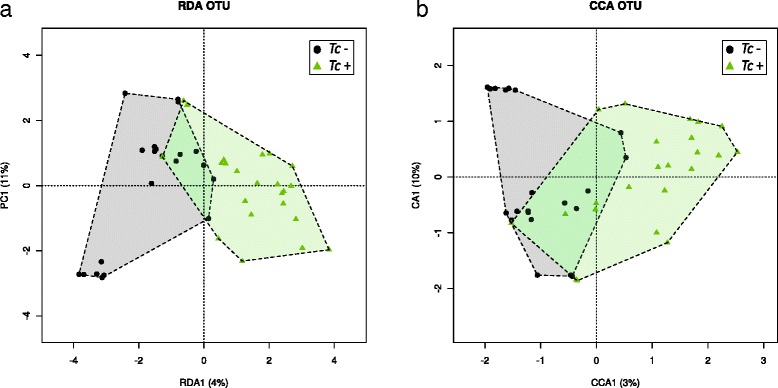
Supervised RDA (**a**) and CCA (**b**) depicting the composition of the faecal microbiota between *Toxocara cati*-positive (*Tc*+) and *T. cati*-negative (*Tc*-) cats

Feline faecal microbial communities were grouped by hierarchical clustering and ordinated by supervised Redundancy Analysis (RDA) and Canonical Correlation Analysis (CCA). Microbial samples from *Tc*+ and *Tc*- cats formed separate clusters (Fig. 3). A significant association between community composition and *T. cati* infection was detected by CCA and RDA analyses (CCA, *P* = 0.030 and RDA, *P* = 0.015, respectively). They were both able to separate samples by infection status (Fig. 3), which suggests a correlation between the latter and the composition of the feline faecal microbiota. The results obtained from the technical replicate of each sample were consistent with the data described above (data available from the corresponding author upon request).

## Discussion

This study investigated, for the first time, the composition of the gut microbiota of feline hosts in presence or absence of patent *T. cati* infection. Our choice to specifically explore the relationships between the latter parasite and the feline commensal flora was motivated primarily by knowledge that *T. cati* is amongst the most prevalent GI parasites of cats worldwide [30], and unequivocal diagnosis of patent infection can be achieved via observation of characteristic dark-brown coloured eggs with thick pitted shells in faecal samples [31]. In addition, while limited information is available on the relationships between the composition of the gut microbiota and infections by selected species of strongylids (i.e. *A. caninum* and *H. contortus*) [22, 23] and enoplids (i.e. *T. suis*) [21], no study, to the best of our knowledge, has analysed the effects of ascarid infections on the make-up of the gut commensal flora of domestic animals.

Besides providing the opportunity to characterise the gut microbiota of cats infected by *T. cati*, this study allowed us to gather an overview of the faecal microbial populations of a relatively homogeneous cohort of feline hosts. According to our observations, the feline gut microbiota is characterised by a significant prevalence of bacteria of the phylum Firmicutes, followed by those of the phyla Proteobacteria, Actinobacteria and Bacteroidetes. These data are in general agreement with the results of previous studies, albeit with some discrepancies linked to the relative contribution of individual phyla of bacteria to the overall composition of the feline commensal flora [32–35]. For instance, the phylum Firmicutes was mostly prevalent in the faecal microbiota of domestic cats fed either a chicken-based extruded diet or raw chicken [36], while bacteria belonging to the Bacteroides group were the most prevalent in the intestinal microbiota of healthy felines fed a commercially available diet whose specific composition was not specified [32]. While these inconsistencies are likely to be linked to dietary differences between the cat cohorts enrolled in our study (fed an identical diet of commercial dry food but allowed to hunt small preys) and those from previous trials [32, 36], methodological differences may also have contributed to these discrepancies. Indeed, while data from our study was generated using Illumina sequencing of the V3-V4 region of the bacterial 16S rRNA, Tun et al. [32] utilised whole metagenome sequencing (i.e. shotgun pyrosequencing, 454) of bacterial DNA; finally, V3-V4 sequences characterised in the investigation by Kerr et al. [36] were generated by pyrosequencing (454). Given the profound differences in detection of bacterial species by whole metagenome shotgun *vs* 16S rRNA amplicon sequencing [37], as well as the fact that the Illumina and 454 technologies are characterised by known differences in sequencing coverage [38, 39], which ultimately affects the overall number of times each OTU is sampled in a metagenomic survey [40], direct comparisons between our results and those from previous studies [32, 36], are unwarranted.

In our study, the gut microbiota of cats harbouring patent infection by *T. cati* was compared with that of cats that were negative for this parasite at the time of sampling. Indeed, it is highly likely that all cats enrolled in this trial had been repeatedly exposed to *T. cati* infections at various stages of their lives, i.e. via (i) infective larvae transmitted by the queens during lactation, (ii) eggs contaminating the environment, and (iii) larvae encysted in the tissues of paratenic hosts. Therefore, we speculate that most if not all *Tc*- cats had had a history of previous infection by this parasite. However, since in this study we aimed to assess the impact that the presence of live *T. cati* exerts on the composition of the feline gut microbiota, previous exposure to the parasite was not taken into account. Comparison of 16S rRNA sequence data between *Tc*+ and *Tc*- cats enrolled in this study by LefSe analysis [29], revealed that, albeit marginally, bacteria belonging to the families *Lactobacillales* and *Enterococcaceae*, both belonging to the phylum Firmicutes, were more abundant in helminth-infected felines. Lactobacilli are members of the lactic acid bacteria, a large group of autochthonous microbes in the gut of humans and animals and that are especially known for their probiotic properties [41]. Interestingly, populations of lactobacilli are expanded in the duodenum of C57BL/6 mice following infection with *H. polygyrus*, while the opposite was observed in the microbiota of infected BALB/c mice, which are relatively resistant to the infection [12]. In addition, a positive correlation was observed between the burden of *H. polygyrus* infection in BALB/c mice and the abundance of lactobacilli in the duodenum of these rodents, which was linked to expansion of Treg cells by the gut-associated lymphoid tissue and production of IL-17A by cells in the mesenteric lymph nodes (MLN) in response to helminth infection [12]. In addition, lactobacilli were markedly increased in the large intestine of C57BL/6 mice chronically infected with *T. muris*, albeit a reduction of frequency of Treg cells in MLN was also observed following infection but prior to the expansion of this bacterial group, which led the authors to hypothesize the existence of different mechanisms of differentiation and development of adaptive immune responses against *T. muris* compared with *H. polygyrus* [15]. Obvious ethical concerns prevented us from investigating the frequency of populations of inflammatory and regulatory cell populations of the gut and associated tissues of the *T. cati* infected cats sampled in our study. However, given the known immune-regulatory properties of a range of nematode parasites [42, 43], as well as those of certain groups of lactobacilli [44], the role of expanded populations of these microbes in helminth-associated modulation of the immune response of human and animal hosts deserves further consideration. Thus far, no information is available on possible associations between bacteria of the genera *Bulleidia* and *Jeotgalicoccus* and infection by GI nematodes. However, the genus *Bulleidia* is frequently associated with the microbiota of the oral cavity of vertebrate hosts [45] while *Jeotgalicoccus* is often isolated from environmental samples [46]. Therefore, the possibility that these genera represent contaminants of the feline faecal samples examined in this investigation cannot be excluded.

In this study, no significant differences in OTU richness and diversity were recorded between *Tc*+ and *Tc*- samples; this finding is in contrast with the results of previous studies of parasite-associated changes in gut microbiota [7, 8, 16, 47], with some exceptions [15, 22]. For instance, helminth-associated increases in microbial diversity were recorded in studies aimed to elucidate the role of the gut microbiota in parasite-induced suppression of inflammation, both in humans with coeliac disease experimentally infected with *N. americanus* [8] and in macaques with idiopathic chronic diarrhoea experimentally infected with *T. suis* [16]. Given that a higher microbial diversity is generally associated with a ‘healthier’ homeostasis of the GI tract [48] and that the gut microbiota of individuals with chronic inflammatory disorders is characterised by a low diversity when compared to healthy individuals [49, 50], these findings led to the hypothesis that the therapeutic properties of parasitic helminths may be due, at least in part, to their ability to promote the restoration of microbial diversity in the gut [8]. The lack of observed differences in microbial richness and diversity between *Tc*+ and *Tc*- cats is likely associated to the relatively small number of animals enrolled in our study, which may have affected the statistical power and thus the ability to detect qualitative and quantitative changes in their gut microbiota. Indeed, whilst the samples analysed were included amongst a much larger number of specimens collected as part of a large multicentric clinical study to assess the efficacy of a new association of parasiticides against feline lungworms [26], a number of ‘filters’ were applied that limited the selection of faecal samples to be subjected to analysis of the microbiota. For instance, cats enrolled in this study originate from a restricted geographical area (i.e. Thessaloniki, Greece) and were fed an identical diet of commercial dry food to minimise the effects of these variables on the composition of their faecal microbiota [51]. In addition, cats diagnosed as infected by GI parasites (i.e. helminths and protozoans) other than *T. cati*, and/or with clinical signs associated with GI disease (e.g. diarrhoea) or any other disease and/or that had been subjected to antibiotic and/or anthelmintic treatment over the 12 months prior to sampling, were excluded, as these elements may have represented significant confounding factors in our analyses [23]. Thus, the limited number of samples analysed prevents us from drawing conclusions on the effects of *T. cati* infection on gut microbial richness and diversity; furthermore, the exact burden of *T. cati* infection in individual cats enrolled in our trial could not be established. The latter is likely to play a major role in investigations of quantitative and qualitative changes in gut microbiota associated to parasite infection, as heavy parasite burdens are likely to be linked to more pronounced shifts in the composition of the commensal flora. Future studies conducted in larger cohorts of felines subjected to a homogeneous diet and for other ascarid nematodes of domestic animals (e.g. experimental infections with *Ascaris suum* in pigs, which would allow an estimate of the parasite burden in each animal host), as well as including samples pre- and post-anthelmintic treatment, are necessary to address these points.

## Conclusions

Data from this study add valuable knowledge to the vast, and yet little explored, research field of parasite-microbiota interactions and will provide a basis for the elucidation of the role such interactions play in pathogenic as well as immune-modulatory properties of parasitic nematodes in both human and animal hosts.

## Additional files

Additional file 1: Dataset S1. Sequence data representing the V3-V4 hypervariable region of the bacterial rRNA amplified from faecal samples from *Toxocara cati*-positive (*Tc*+) and *T. cati-*negative (*Tc*-) cats, and taxonomic assignment. (XLSX 3501 kb)
Additional file 2: Figure S1. Hierarchical clustering heatmap of the composition of the faecal microbiota phyla of *Toxocara cati*-positive (*Tc*+) and *T. cati-*negative (*Tc*-) cats. Dendrograms at the top of the heatmap indicate relationships between samples. Colour intensity represents the relative abundance of sequences representing the corresponding bacterial family in each sample. (PDF 63 kb)

## Abbreviations

16S rRNA: 16S ribosomal RNA gene
BP: Base pairs
CCA: Canonical Correlation Analysis
DNA: Deoxyribonucleic acid
EPG: Eggs per gram of faeces
FEC: Faecal egg counts
GI: Gastrointestinal
LDA: Linear discriminative analysis
MLN: Mesenteric lymph nodes
NCBI: National Centre for Biotechnology Information
OTU: Operational Taxonomic Unit
PCR: Polymerase chain reaction
QIIME: Quantitative Insights Into Microbial Ecology
RDA: Redundancy Analysis
RNA: Ribonucleic acid
*Tc*-: *Toxocara cati*-negative
*Tc*+: *Toxocara cati*-positive
