# Supplementary figures and images for: Helminth infections and gut microbiota – a feline perspective
Source: Parasit Vectors. 2016 Dec 3;9:625. doi: 10.1186/s13071-016-1908-4 (PMC5135779; doi:10.1186/s13071-016-1908-4)

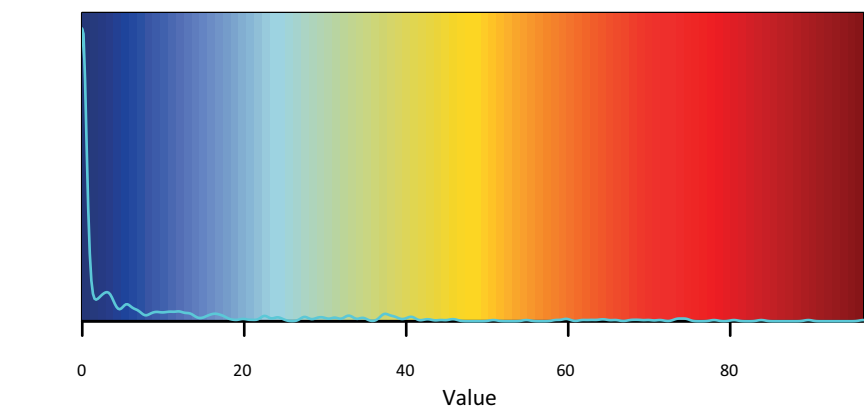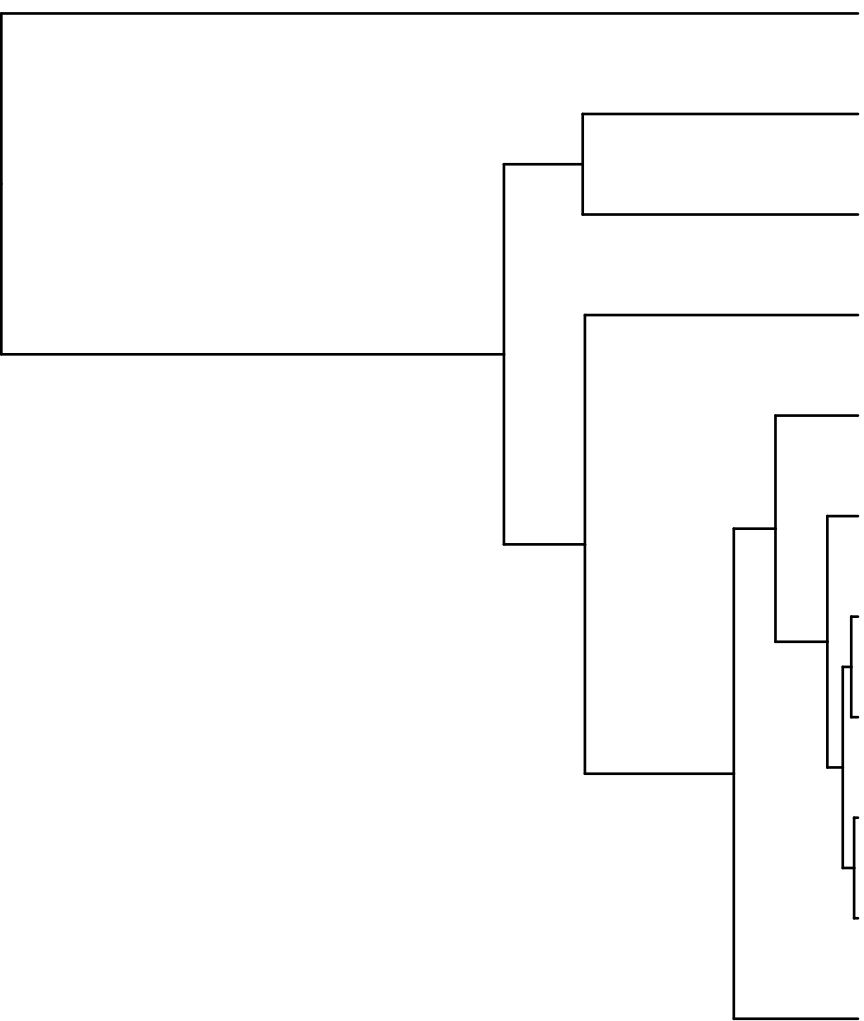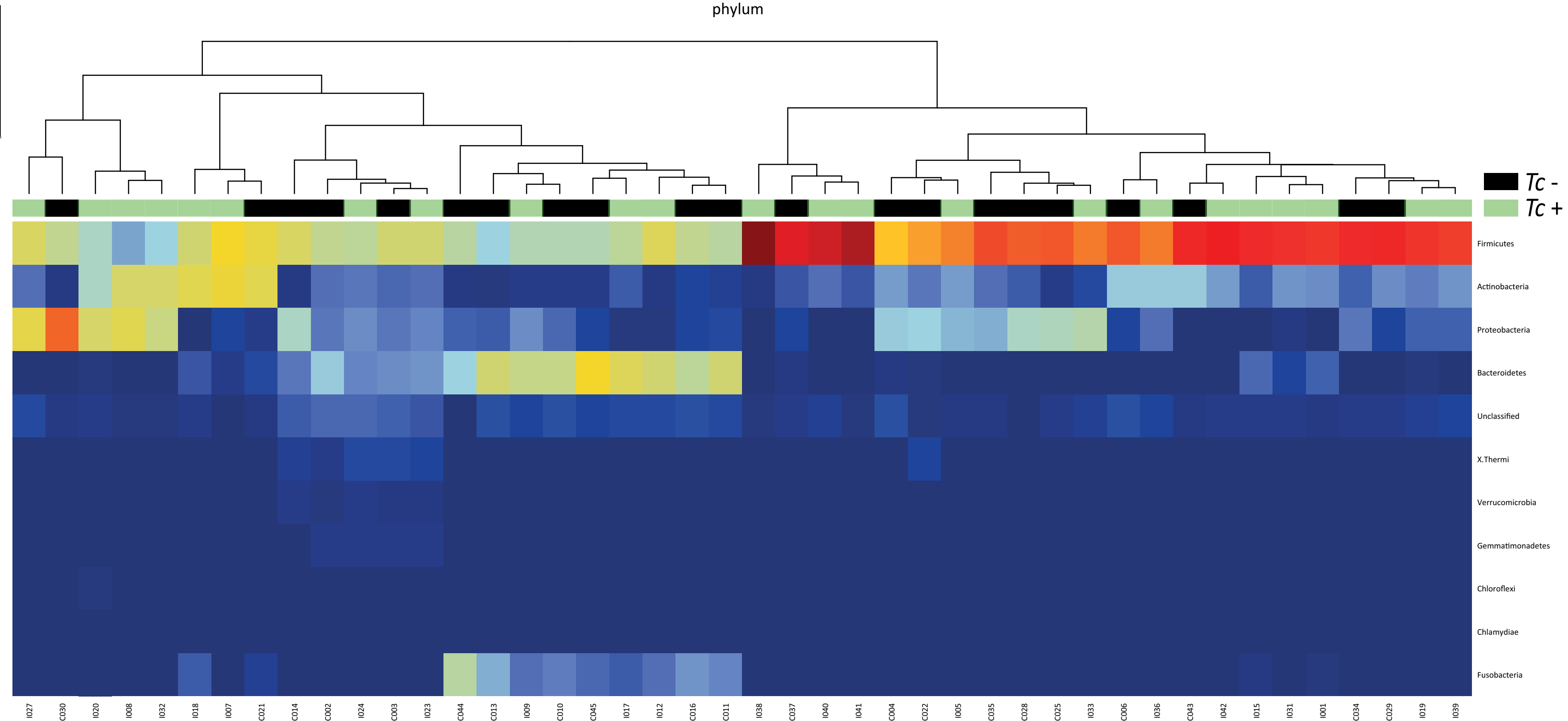

Supplement: Additional file 2: Figure S1. — Hierarchical clustering heatmap of the composition of the faecal microbiota phyla of Toxocara cati-positive (Tc+) and T. cati-negative (Tc-) cats. Dendrograms at the top of the heatmap indicate relationships between samples. Colour intensity represents the relative abundance of sequences representing the corresponding bacterial family in each sample. (PDF 63 kb) [file 13071_2016_1908_MOESM2_ESM.pdf]
